# Ceramic-in-Polymer Composite Solid Electrolyte Enabled by Metal–Sulfur Interactions with Enhanced Li-Ion Conductivity

**DOI:** 10.1021/acsaem.5c01010

**Published:** 2025-06-26

**Authors:** Beibei Jiang, Zhantao Liu, Hailong Chen, Yiming Zhang, Vladimir V. Tsukruk, Junjun Hu, Zhiming Qiang, Manika Tun Nafisa, Benjamin Klein, Tara Joshi

**Affiliations:** a Department of Electrical and Computer Engineering, 15617 Kennesaw State University, Marietta, Georgia 30060, United States; b Department of Mechanical Engineering, 1372 Georgia Institute of Technology, Atlanta, Georgia 30332, United States; c Department of Materials Science and Engineering, 1372 Georgia Institute of Technology, Atlanta, Georgia 30332, United States

**Keywords:** ceramic-in-polymer composites, photopolymerization, zirconium/sulfur, lanthanum/sulfur, coordination, electron-transfer reaction, solid electrolyte, ionic conductivity

## Abstract

Ceramic-in-polymer composite solid electrolytes (SEs) show great potential for meeting the high-performance requirements of all-solid-state batteries (ASSBs) due to the combined benefits of easy processability, tunable Li-ion conductivity, wide electrochemical window, and facile interfacial contact with the Li-metal anode. However, their Li-ion conductivity remains lower than that of the pure ceramic phase, which can be attributed to the highly resistive ceramic/polymer interphase. In this paper, we introduced sulfur-containing functional groups through a less-explored metal/sulfur interaction strategy, enabling simultaneous modification of the polyethylene glycol diacrylate (PEGDA) polymer scaffold and the Li_6.4_La_3_Zr_1.4_Ta_0.6_O_12_ (LLZO) ceramic surface. We elucidated the nature of metal/sulfur interactions, i.e., the preferential coordination interaction between Zr and sulfur, as well as electron-transfer reactions from sulfur to Zr and La atoms. In addition, we unraveled the mechanisms of metal/sulfur interaction-enabled *in situ* photopolymerization of the PEGDA scaffold and developed a layer-by-layer method that exploits metal/sulfur interactions for manufacturing sulfur-modified LLZO-in-PEGDA composites. This dual-modification strategy effectively promotes Li-ion transport at both LLZO/LLZO and LLZO/PEGDA interphases, resulting in enhanced ionic conductivity and lower activation energy. As a result, the LLZO-in-PEGDA composite exhibited a high conductivity of 5.1 × 10^–4^ S cm^–1^, exceeding the vendor-reported conductivity of pure LLZO. In addition, the sulfur-modified LLZO-in-PEGDA composites exhibited improved toughness and stretchability, suggesting the potential dual role as a protective layer for electrode materials. The metal/sulfur-interaction-enabled dual modification offers a promising strategy that can be broadly applied to the rational design of ceramic/polymer composite materials.

## Graphical abstract

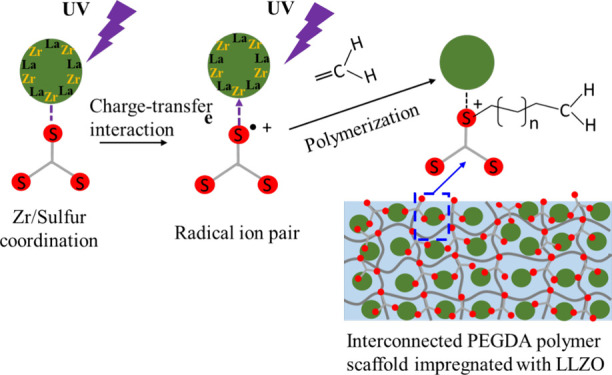

## 1 Introduction

Li-metal ASSBs, which utilize nonflammable SEs together with a Li metal anode, are considered the ultimate solutions for future energy storage due to their potential of combining high-energy density, high power density, superior safety, and extended lifespan. However, the commercialization of Li-metal ASSBs remains limited, largely due to the challenges associated with SEs, including insufficient Li-ion conductivity, limited electrochemical stability, poor compatibility with Li-metal, inadequate mechanical strength, and poor interfacial contact with electrodes.^1–5^ Developing high-performance SEs with these key properties is essential for advancing the Li-metal ASSB technology.

SEs are currently classified into three main categories: polymer, ceramic, and composites.^6^ Polymer electrolytes, such as the extensively studied poly­(ethylene oxide) (PEO)-based Li-ion conductors, typically exhibit good processability and facilitate a facile ionic interface between the electrolyte and electrodes.^7^ Li-ion transport in PEO is more favorable in the amorphous phase than in the crystalline phase, with ionic conductivity ranging from 10^–6^ to 10^–8^ S cm^–1^ at 20 °C for amorphous PEO and below 10^–8^ S cm^–1^ for crystalline PEO.^1,5,8^ Ceramic electrolytes, such as garnet-type Li_7_La_3_Zr_2_O_12_ (LLZO) and its doped variants, exhibit higher ionic conductivity (i.e., >10^–4^ S cm^–1^ at 20 °C) and a near-perfect transference number (i.e., ∼1.0).^9–11^ The garnet-type LLZO family is currently the only class of ceramic electrolytes that shows adequate stability against reduction by Li.^12^ However, they are very brittle, making them difficult to process and challenging to maintain a facile ionic interface with the electrodes.^13^ Ceramic/polymer composite electrolytes, which combines the advantages of both components, demonstrate a good combination of easy processability, tunable ionic conductivity (10^–4^–10^–5^ S cm^–1^),^14–16^ and high mechanical modulus.^17^ The “classical” ceramic-in-polymer composites, where the ceramic fillers are dispersed within a polymer network, inherit the easy processability of polymers and are suitable for wide applications. However, their ionic conductivity remains lower than that of the pure ceramic phase,^1^ primarily due to the high ionic resistance at the ceramic/polymer interphase and within the polymer scaffold.^18^ This limitation necessitates careful modulation of factors such as composite composition, polymer scaffold architecture, and interfacial properties.^5,8^

Modifying the polymer scaffold or ceramic surface with functional groups has recently emerged as a promising strategy to lower the intrinsic ceramic/polymer interfacial barrier, enabling the construction of highly conductive ceramic/polymer interphases.^19^ For example, a polyacrylonitrile (PAN)-modified Li_6.4_La_3_Zr_1.4_Ta_0.6_O_12_ (LLZTO) surface layer has been achieved through the strong coordination interaction between La atoms in LLZTO and nitrile groups in PAN. The preferential adsorption of the nitrile groups on the surface of LLZTO forms a polymer protective layer on the electrolyte, providing fast Li-ion transport at the interface, delivering stable ionic conductivity up to 10^–4^ S cm^–1^ and high Li-ion transference number (0.66).^20,21^ Besides nitrile group, other commonly used functional groups for modifying polymer scaffolds or ceramic fillers include carboxyl, hydroxyl, pyridine, and pyrrole groups.^20^

Sulfur-based functional groups exhibit “chameleonic” behavior in their chemical, redox, and electronic properties, as well as in their coordination behavior, due to unique features of sulfur species, such as high polarizability, large negative charge, and coordination versatility.^22^ The interaction of sulfur-based functional groups with metal ions has been extensively studied in metal–organic frameworks (MOFs) for various applications, including gas separation, catalysis, and lithium–sulfur batteries.^23–25^ However, their potential as an interfacial modification strategy for enhancing Li-ion transport at the ceramic/polymer interface in composite electrolytes remains largely unexplored. Through metal–sulfur coordination interactions, we fabricated ceramic-in-polymer composites consisting of Li_6.25_Al_0.25_La_3_Zr_2_O_12_ (LLZO) ceramic fillers encapsulated within cross-linked PEGDA polymer scaffold (denoted as LLZO-in-PEGDA). The cross-linked PEGDA was chosen as polymer scaffold for its key advantages: (1) higher ionic conductivity than PEO-based due to its amorphous structure;^26^ (2) ability to support high ceramic loading due to its cross-linked architecture; (3) capable of incorporating highly conductive ionic liquid (IL) in the composite while maintaining solid form; and (4) suitable for *in situ* sulfur modification via thiol–ene click reaction. We identified unique metal–sulfur interactions between Zr/La ions in LLZO and sulfur-containing thiol groups present on the surface of LLZO. We further elucidated the polymerization mechanisms driven by metal–sulfur interactions and developed a layer-by-layer fabrication method that exploits these interactions to construct rationally designed LLZO-in-PEGDA composites with precise control over composition, structure, homogeneity, and LLZO/PEGDA interphases. The rationally designed composite exhibits lower activation energy and an enhanced ionic conductivity of 5.1 × 10^–4^ S cm^–1^, comparable to or even exceeding that of pure LLZO. Furthermore, the rationally designed LLZO-in-PEGDA composites exhibits improved toughness and stretchability, suggesting the potential dual role as a protective layer for electrode materials. This modification strategy through metal/sulfur interactions holds promise as a general strategy for the design and synthesis of advanced ceramic/polymer composite materials.

## 2 Materials and Methods

### 2.1 Materials

Dichloromethane (CH_2_Cl_2_, Sigma-Aldrich), trimethylolpropane tris­(3-mercoptopropianate) (TT, Sigma-Aldrich), poly­(ethylene glycol) diacrylate (PEGDA, Mn = 700g/mol, Sigma-Aldrich); 1-ethyl-3-methylimidazolium bis­(trifluoromethylsulfonyl)­imide (EMIM-TFSI, Sigma-Aldrich), bis­(trifluoromethane) sulfonamide lithium (LiTFSI, Sigma-Aldrich), benzoin methyl ether (Photoinitiator), Al-doped, Li_6.25_Al_0.25_La_3_Zr_2_O_12_ (LLZO, cubic garnet, 500 nm, bulk ionic conductivity = 5 × 10^–4^ S cm^–1^, MSE supplies), and TiO_2_ nanoparticles (anatase, Fisher scientific) were used without further purification.

### 2.2 Preparation of TT-Treated LLZO to Investigate Metal/Sulfur Interactions

0.33g LLZO nanoparticles (0.33g) were dispersed in 5 mL of dichloromethane and stirred thoroughly for 30 min. Then, thiol-containing TT was added dropwise to the solution under continuous stirring and allowed to stir for 3 h. Different amounts of TT were used, with the sulfur/Zr atomic ratio varying from 2:1 to 12:1. After stirring for 3 h, the TT-treated LLZO were purified in dichloromethane by centrifuging to remove any uncoordinated TT molecules. The TT-treated LLZO was characterized by FTIR, Raman, and XPS to elucidate the metal–sulfur interactions.

### 2.3 Layer-by-Layer Fabrication of Sulfur-Containing LLZO-in-PEGDA Composites via Metal/Sulfur Interactions

The LLZO-in-PEGDA composites consisting of an interconnected PEGDA polymer scaffold loaded with LLZO as active materials were prepared by the layer-by-layer photopolymerization process. To investigate the effect of various intrinsic interphases on Li-ion conductivity within the composites, IL, such as EMIM-TFSI, was also introduced in selected LLZO-in-PEGDA composites. In the beginning, precursor slurries were prepared by mixing LLZO nanoparticles, the PEGDA monomer, LiTFSI, and/or IL in light-protected glass vials. These mixtures were magnetically stirred for over 24 h to achieve a series of homogeneous precursor mixtures containing different weight percentage (wt %) of active materials. For precursor slurries with high wt % LLZO and 0 wt % IL, CH_2_Cl_2_ solution was added to adjust the slurry viscosity. The slurries were then coated on glass or Cu foil substrates using the standard tape-casting coater to obtain the initial wet precursor film (∼50 μm). Subsequently, the TT film was coated above the initial LLZO-containing precursor film by the same tape-casting process using CH_2_Cl_2_ diluted TT solution (v/v = 1:1). The immediate contact of a sulfur-containing TT layer with an LLZO-containing precursor layer will lead to immediate solidification of the composite film upon UV illumination, without adding any exogenous photoinitiator. The solidified film was exposed to UV illumination (254 nm) for 5 min, leading to a uniform film with some unreacted and active thiol groups on the surface. The above process was repeated 3–6 times for controlling the film thickness. Finally, the resulting multilayered films were vacuum-dried under 60 °C overnight. The films can be easily peeled off from the substrates to obtain free-standing films.

### 2.4 Fabrication of Sulfur-Free LLZO-in-PEGDA Composites (as Control Samples)

For studying the effect of metal–sulfur interactions, control samples without adding sulfur-containing molecules (i.e., TT) were fabricated by a similar approach with some minor differences. First, when preparing the precursor slurries, benzoin methyl ether (1 wt % of the total weight of PEGDA precursors) was added as photoinitiator for initiating the photopolymerization process. Then, the precursor slurries were coated on glass or Cu foil substrates by the same tape-casting process. Finally, the multilayered wet precursor films were exposed to UV illumination for 20 min to obtain solid films and finally dried under vacuum at 60 °C overnight. The films can also be easily peeled off from the substrates to obtain free-standing films.

## 3 Characterization

The Fourier-transform infrared spectroscopy (FTIR) for the free-standing LLZO-in-PEGDA composite films was collected using a Thermo Nicolet ATR FTIR from 500 to 4000 cm^–1^ at room temperature. The kinetics for the photopolymerization process was monitored by the acrylate group (1640 cm^–1^, C=C twisting vibration band) and thiol group (2560 cm^–1^, S–H twisting vibration band). To further detect the relatively weak coordination bonding between metal ions and thiol groups, TT-treated LLZO powders and LLZO-in-PEGDA composite films were characterized by JASCO FTIR-6x in Far-IR range from 500 to 20 cm^–1^ at room temperature.

### 3.1 Raman Characterization

Raman spectra were collected with a Renishaw micro-Raman system under the excitation of 532 nm, with 1800 lines/mm holographic grating and ×100 magnification.

### 3.2 XPS Characterization

The X-ray photoemission spectroscopy (XPS) data of TT-treated LLZO powders were obtained through an X-ray photoelectron spectrometer (Thermo Scientific K-Alpha) provided by the MSE Analytical Service.

### 3.3 Electrochemical Characterization

The ionic conductivity of the composite SEs was measured by a Gamry Interface 1010E potentiostat, where the samples were sandwiched between two polished stainless-steel electrodes. The AC impedance was measured in the frequency range from 2 MHz to 1 Hz at 10 mV amplitude. To determine the temperature dependence of ionic conductivity, the samples were placed in a temperature-controlled chamber set between 20 and 80 °C. Two heat/cool cycles were applied for each 10 °C change. After reaching the set temperature, the samples were allowed to rest for 1 h before collecting AC impedance. The ionic conductivity (σ) of the composites at each temperature was calculated by eq 1:

$$\sigma =\frac{d}{R_{b}\times A}$$ 1

where *d* is the film thickness, and *R_b_* is the film bulk resistance and was taken from the minimum of the phase angle from the electrochemical impedance spectroscopy, which is the same value with the semicircle intersects with the abscissa on the Nyquist impedance plot. *A* represents the effective electrode surface area.

### 3.4 Scanning Electron Microscopy (SEM)

The morphology of the composites was analyzed by SEM (Phenom XL G2 SEM SOP).

### 3.5 Mechanical Tension Test

Tension mechanical tests were conducted using a Shimadzu EZ-SX tester at a crosshead speed of 0.5 mm/min. Three samples were collected for each test.

### 3.6 Cyclic Voltammogram (CV)

CV was used to evaluate the electrochemical stability of the LLZO-in-PEGDA composite electrolytes. The CV tests were performed on Cu|Composite|Li cells in the potential window of −0.1 to 4 V for 6 cycles at a scan rate of 0.01 V/s by using BioLogic electrochemical workstation.

## 4 Results and Discussion

### 4.1 Interactions between LLZO and Thiol Group (−SH)

The direct addition of thiol-containing molecules (i.e., TT) into the precursor solution of LLZO, PEGDA monomer, and CH_2_Cl_2_ solvent resulted in immediate solidification of the mixture within 5 s (see Figure S1 and supplementary video), leaving no room for controlling the composition, structure, and homogeneity of the resulting composites, if using the classical photopolymerization approach. In addition, the solidified films cannot be dissolved in common solvents, suggesting the formation of strong bonds or the polymerization of PEGDA monomer (see Figure S2). Such an immediate solidification reaction only occurred when TT and LLZO were both present in the mixture. When LLZO was replaced with inactive nanofillers, such as SiO_2_ and Al_2_O_3_, the solution remained in a liquid state. When PEGDA monomer is absent in the mixture, the solution also remained in a liquid state.

To understand whether the interaction between LLZO and TT has triggered the polymerization of PEGDA, the solidified films were subject to a time-dependent FTIR characterization under UV illumination or controlled light protection conditions; see results in Figure 1a–c. After the solidified films formed (i.e., *t* = 0 min), the characteristic carbon–carbon double bond (−C=C−) of the acrylate at 1640 and 810 cm^–1^ was still present,^27–29^ suggesting the presence of unreacted PEGDA monomers. However, as UV exposure time increases, both peaks vanished (Figure 1b), confirming the −C=C– bonds participated in the reaction, forming an interconnected PEGDA polymer network. In contrast, when the solidified film was kept under light protection with no UV illumination, the peaks for −C=C– bonds weakened but still existed (Figure 1a), demonstrating UV exposure is essential for initiating the polymerization of PEGDA. Notably, the same mixture with an external photoinitiator (Figure 1c) exhibited the same −C=C– bond behavior as the mixture without a photoinitiator (Figure 1b). All of the above suggest that the interaction between LLZO and thiol groups plays a role analogous to that of a photoinitiator in initiating the thiol–ene click polymerization of PEGDA.

**Figure 1:**
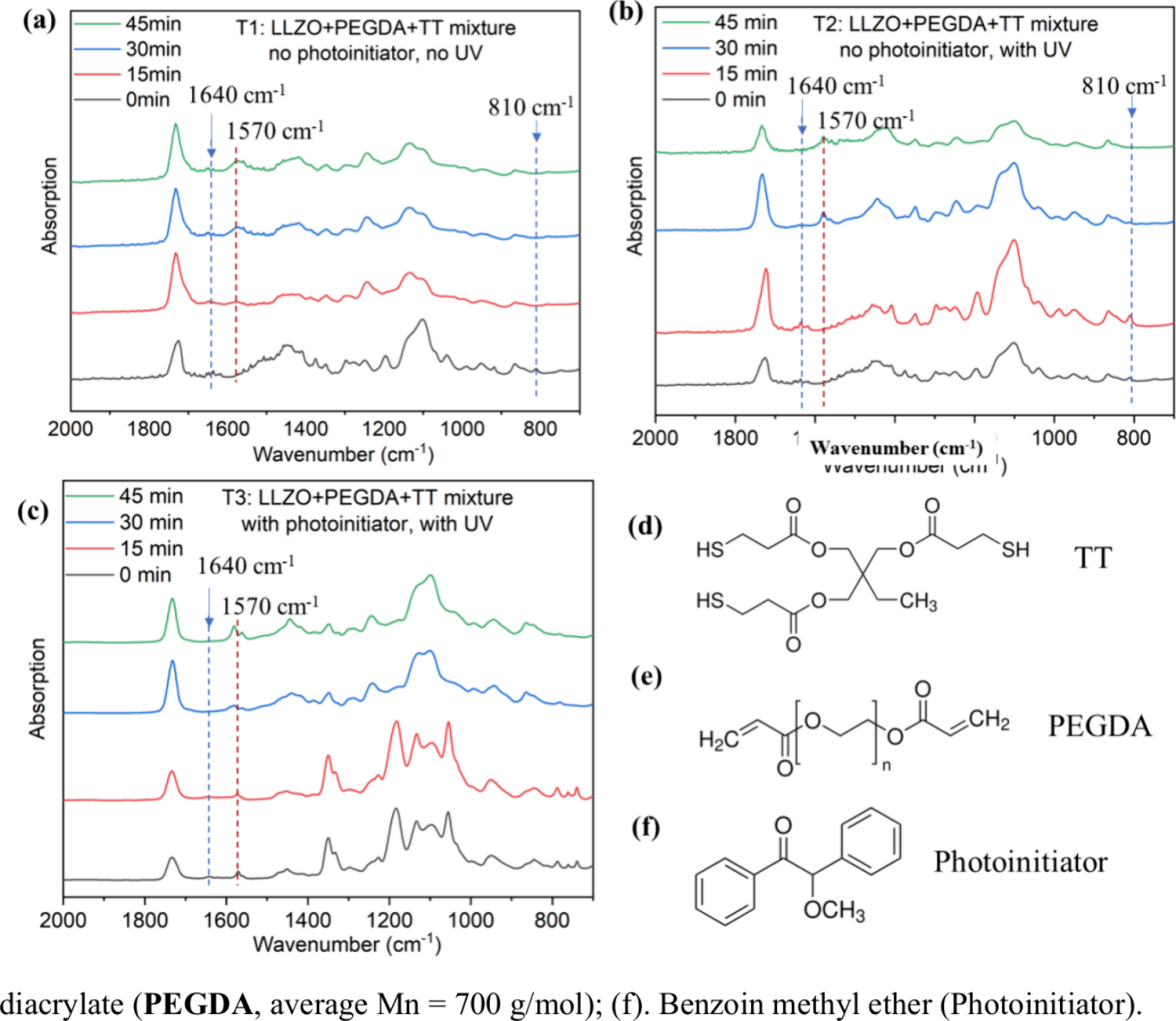
(a) Time-dependent FTIR characterization for the LLZO/PEGDA/TT mixture under (a) no photoinitiator and no UV exposure; (b) no photoinitiator and with UV exposure; (c) with photoinitiator and with UV exposure and chemical structures for materials used in this study; (d) trimethylolpropane tris­(3-mercoptopropianate) (TT); (e) PEGDA (average Mn = 700 g/mol); (f) benzoin methyl ether (photoinitiator).

In the UV-initiated thiol–ene click reaction between PEGDA and TT, the photoinitiator generates radicals upon UV exposure, which is key for initiating polymerization. The generated radicals abstract hydrogen from thiol groups (−SH), forming thiyl radicals (R–S^•^).^29^ The thiyl radicals then attack the ene groups (i.e., −C=C−), generating carbon radicals. The carbon radicals can react with other −C=C– via the conventional chain-growth mechanism or react with thiol groups to regenerate thiyl radicals, facilitating further thiol–ene addition reactions through the step-growth mechanism.^29^ In a study on MOF-based thiyl-radical catalysis consisting of Zr_6_ clusters and a bridging ligand featuring four thiol groups next to two carboxylic binding sites, thiyl radicals (R–S^•^) were generated due to the accelerated electron transfer from the thiol groups to Zr_6_ cluster upon photon activation,^30^ which suggests that thiyl radicals (R–S^•^) can also be produced by electron-transfer processes. Given the well-established understanding of the thiol–ene click reaction and prior evidence of thiol-Zr electron-transfer interaction, it is reasonable to hypothesize that the sulfur-containing thiol groups experience both coordination and electron-transfer interactions with metal ions in LLZO, facilitating the generation of thiyl radicals and initiating the photopolymerization of PEGDA monomers upon UV illumination.

To elucidate the hypothesis mentioned above that photopolymerization is initiated by interactions between LLZO and thiol groups, TT-treated LLZO was prepared as described in section 2.2 and the detailed characterization results are present in Figure 2a–f. Figure 2a shows Raman spectroscopy of LLZO before and after TT functionalization. For raw LLZO, the bands at the lower end of the spectrum (100–150 cm^–1^) correspond to the vibration of La cations.^31^ The bands between 200 and 300 cm^–1^ correspond to the vibration of LiO_6_ octahedra, while the bands between 300 and 500 cm^–1^ correspond to the LiO_4_ tetrahedra.^32^ The intense band at 1090 cm^–1^ is attributed to the symmetric stretching vibration of Li_2_CO_3_.^33,34^ Notably, the band near 639 cm^–1^ for raw LLZO is attributed to the stretching vibration of Zr–O bond in ZrO_6_ octahedra and thus is sensitive to vibration in the Zr coordination.^33,35^ As S/Zr ratio increases, the peak at 639 cm^–1^ for Zr–O bond systematically shifts to a higher wavenumber (645, 650, 652, and 656 cm^–1^), suggesting that the Zr–O bonds become shorter upon coordinating with sulfur.^36^ The shifting of the Zr–O bond is more significant than that of other peaks, suggesting that sulfur shows more preference to coordinate with Zr^4+^ than other metal ions in LLZO. To further confirm sulfur coordination with Zr, TT-treated ZrO_2_ samples were analyzed by Raman spectroscopy (Figure S3). Several distinct vibrational bands were observed at 162, 174, 318, 328, 364, 490, and 560 cm^–1^ for raw ZrO_2_, consistent with the monoclinic phase of ZrO_2_ (M) with slight deviation in peak positions.^37,38^ As the S/Zr ratio increases, these peaks also shift progressively to higher wavenumbers. This systematic shift of Zr–O bond toward higher wavenumber in both LLZO and ZrO_2_ confirms the preferential coordination between Zr ions and sulfur.

**Figure 2:**
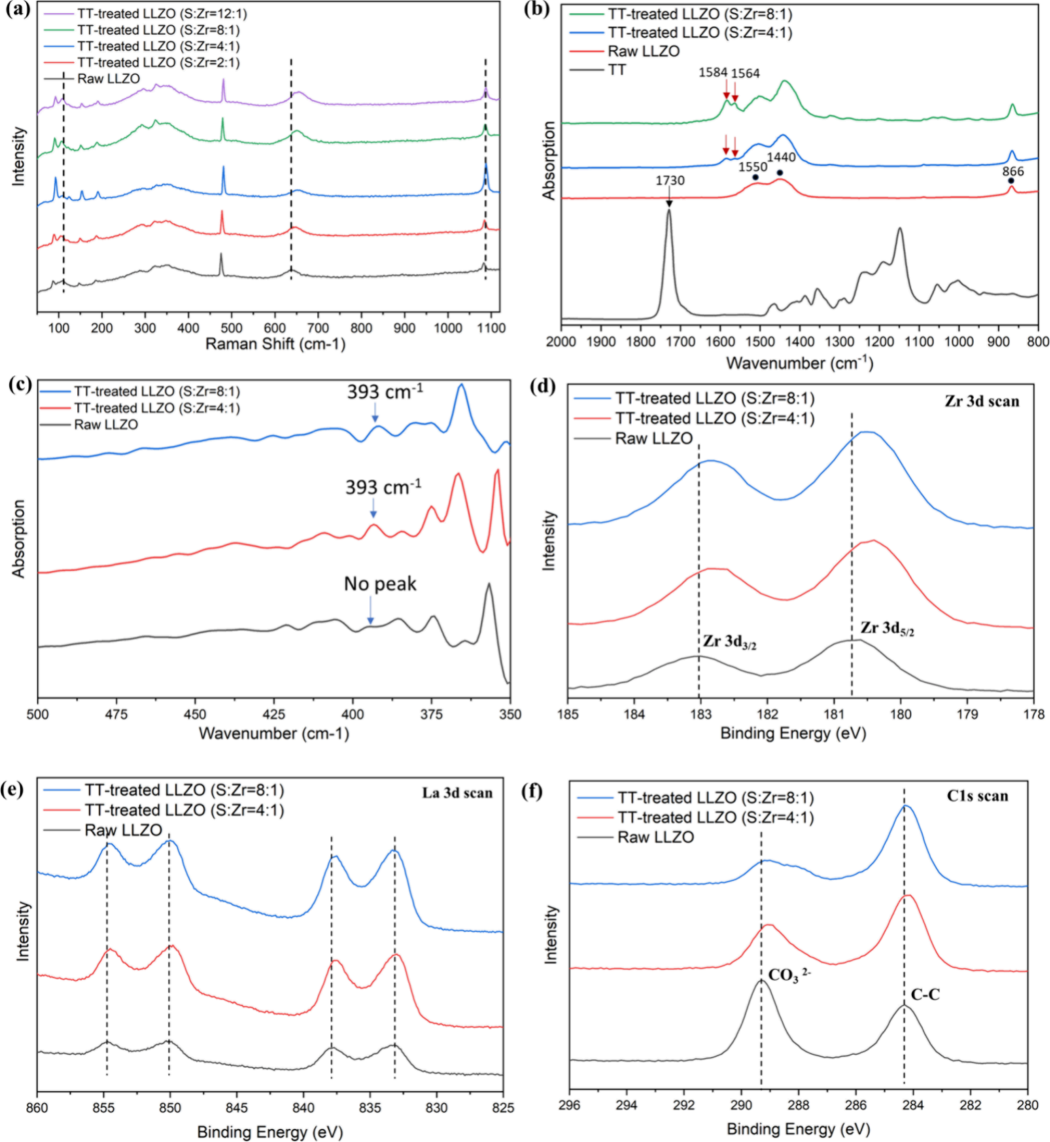
(a) Raman spectra; (b,c) far-IR FTIR spectra; and (d–f) XPS spectra of LLZO before and after treatment with sulfur-containing TT at different S to Zr ratio.

Figure 2b,c shows the Far-IR FTIR spectra of LLZO before and after TT functionalization. In Figure 2b, characteristic peaks at 866, 1440, and 1550 cm^–1^ correspond to carbonyl group (−C=O) vibration in −CO_3_, indicating the presence of Li_2_CO_3_ on the surface of LLZO.^39,40^ Upon TT treatment, new doublet peaks emerge at 1564 and 1584 cm^–1^, with their peak intensity increasing as the S/Zr ratio increases. This doublet peak is consistent with the new peak observed near 1570 cm^–1^ in Figure 1a,b. As this new peak takes some time to form (as seen in Figure 1a), this new peak could be assigned to the coordinated −C=O group (in ester group of TT) with Li_2_CO_3_ on LLZO.^41^ Figure 2c extends the FTIR analysis into the Far-IR regime (400–20 cm^–1^), which is commonly used to detect relatively weak bonds, such as coordination bonding.^42^ A new peak at 393 cm^–1^ appears in TT-treated LLZO samples, which could be attributed to the formation of a Zr–S coordination bond. Similar Zr–S coordination bonds have been observed in other studies, such as a porous coordination polymer gel based on Zirconium­(IV) and 2-thiobarbituric acid, where a corresponding Zr–S peak was detected at 323 cm^–1^.^43^ The shift may arise from differences in the precursor materials and local chemical environments.

The change in chemical environment after coordination interaction was probed by X-ray photoelectron spectroscopy (XPS), as shown in Figure 2d–f. In the partial scan of Zr 3d (Figure 2d), two peaks appeared at 180.76 and 183.05 eV in raw LLZO, corresponding to the binding energies of Zr 3d_5/2_ and Zr 3d_3/2_, respectively.^44,45^ In TT-treated samples, the peaks shifted to lower binding energy by 0.28 and 0.27 eV respectively. A similar phenomenon was observed in the partial scan of La 3d (Figure 2e), where the two sets of La 3d peak at binding energy of 854.75, 850.25, 837.86, and 833.28 eV appeared in raw LLZO. After TT functionalization, these peaks slightly shifted to lower binding energies by 0.23, 0.23, 0.24, and 0.18 eV, respectively. The lower shifting of the binding energy indicates an increase in electron density around Zr and La atoms, which could be donated by sulfur atoms assisted by the short distance between them due to coordination bonding between Zr and Sulfur.^29^ This electron-transfer interaction between sulfur in thiol groups and metal ions in LLZO requires photoactivation via UV illumination (as demonstrated in Figure 1a), leading to the formation of thiyl radicals that can generate the photopolymerization observed here.^29^

### 4.2 Metal/Sulfur Interaction-Initiated Polymerization: Mechanism

Based on the above observations, we proposed a synthetic mechanism to explain the formation of LLZO-in-polymer composites facilitated by the metal/sulfur interactions, as depicted in Figure 3. The mechanism involves two key interactions between metal ions and sulfur: coordination interaction between Zr and sulfur, as well as electron-transfer reactions from sulfur to Zr and La atoms.^46^ The strong Zr/Sulfur coordination interaction enables sulfur-containing TT molecules to bind to the LLZO surface, functioning as surface ligands. Each TT molecule, possessing three thiol groups, also serves as a bridging agent, connecting LLZO nanoparticles and forming an interconnected LLZO network. This network is capable of absorbing liquid solutions, such as monomers and IL, as seen in this work, resulting in a mixture that appears solid. In addition, the local environment due to surface ligands can change the binding, kinetics, redox potentials, and proton and electron transfer without a direct metal interaction.^47^

**Figure 3:**
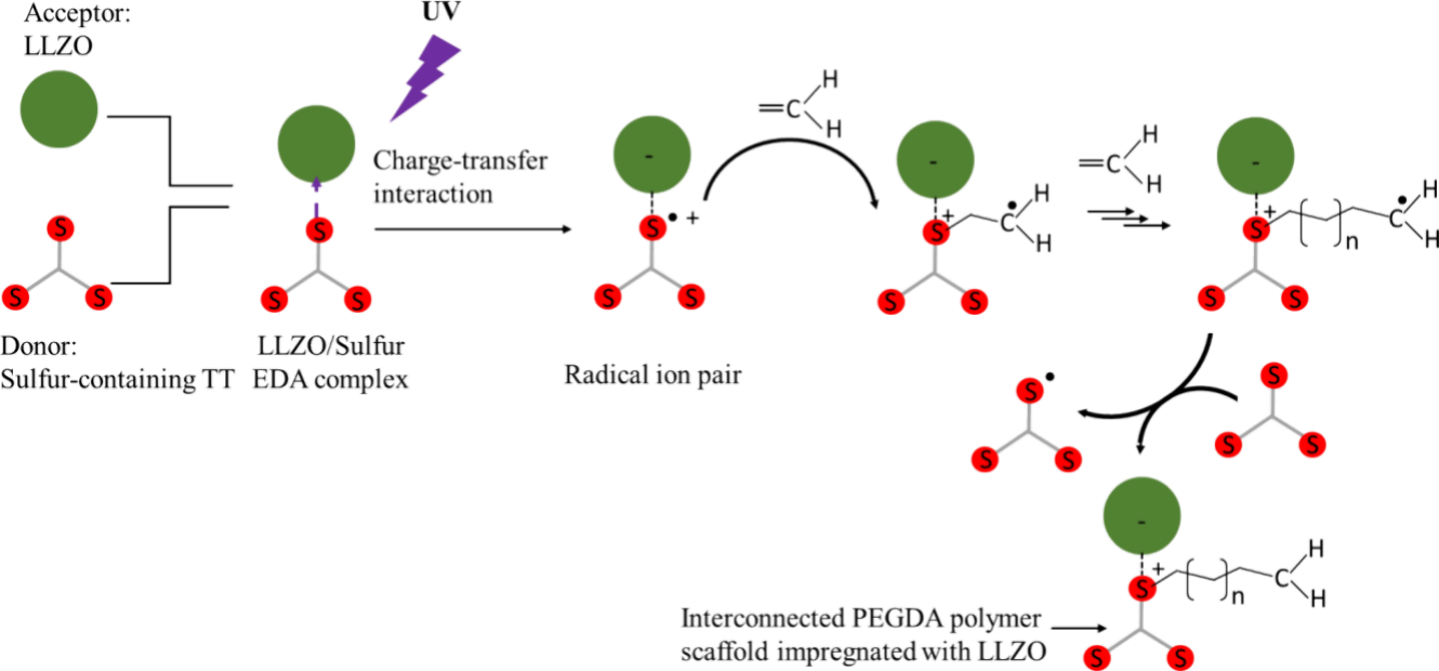
General scheme illustrating the metal/sulfur interaction-facilitated photopolymerization process for fabricating LLZO-in-polymer composites. For simplicity, the scheme depicts only the interactions between each Zr atom and a single sulfur atom.

The electron transfer from sulfur to Zr and La atoms, on the other hand, is a more intriguing aspect of metal/sulfur interactions. Recent studies have revealed the electron donor–acceptor (EDA) photoactivation between Zr-containing MOF and sulfonium, generating open-shell radical species for cross-coupling reactions.^46^ As illustrated in Figure 3, the UV light excitation triggered the intramolecular electron-transfer event between sulfur donor and LLZO acceptors, forming the LLZO/sulfur EDA complex. Upon donating one electron, the thiol groups in TT decompose to generate radical intermediates, such as thiyl radical (−S·). The thiyl radical then attacks a carbon–carbon double bond in the acrylate groups of the monomer, forming carbon radical (−C·). The carbon radical subsequently reacts with the abundant acrylate groups via a free-radical chain-growth mechanism, forming interconnected PEGDA polymer scaffold. Additionally, the carbon radical may abstract hydrogen from a free thiol functional group, regenerating thiyl radicals that can reinitiate the thio-ene addition reaction via a step-growth mechanism. The latter reaction is not depicted in the schematics, as the free thiol functional group is likely limited due to the participation of the strong Zr/sulfur coordination. For simplicity, the scheme depicts only the interactions between each LLZO and one thiol group. In reality, each LLZO may coordinate with multiple thiol groups.

## 5 Layer-by-Layer Fabrication of LLZO-in-PEGDA Composite SEs via Metal/Sulfur Interactions

### 5.1 Characterization of LLZO-in-PEGDA Composites

To elucidate the impact of metal–sulfur interactions on Li-ion conductivity, a layer-by-layer fabrication method was developed, as shown in Figure 4. A thin initial layer was formed by applying precursor slurries with varying LLZO to PEGDA weight ratios onto preferred substrates via the doctor blade coating method. In this process, LLZO nanoparticles were uniformly dispersed within the viscous monomer thin film. When the second layer of TT was applied over the first layer, the strong Zr/S coordination facilitated the “bridging” of LLZO by TT ligands. Each TT molecule, containing three thiol groups, coordinated with metal ions from different LLZO nanoparticles, promoting interparticle connections. This process led to the formation of interconnected LLZO scaffolds impregnated with PEGDA monomers, resulting in the “solidification” of the film while preserving its homogeneous composition. The subsequent UV irradiation process triggered the intramolecular single-electron-transfer event between sulfur donor and LLZO acceptor due to the short distance between them, leading to the generation of thiyl radical (−S·) and polymerization of PEGDA via the chain-growth mechanism mentioned above. This process leads to the formation of cross-linked PEGDA polymer scaffolds impregnated with LLZO fillers. Notably, the LLZO filler is also anchored to the polymer scaffold due to the metal/sulfur interactions. The above process was repeated multiple times to achieve the desired thickness, making it suitable for use as SEs.

**Figure 4:**
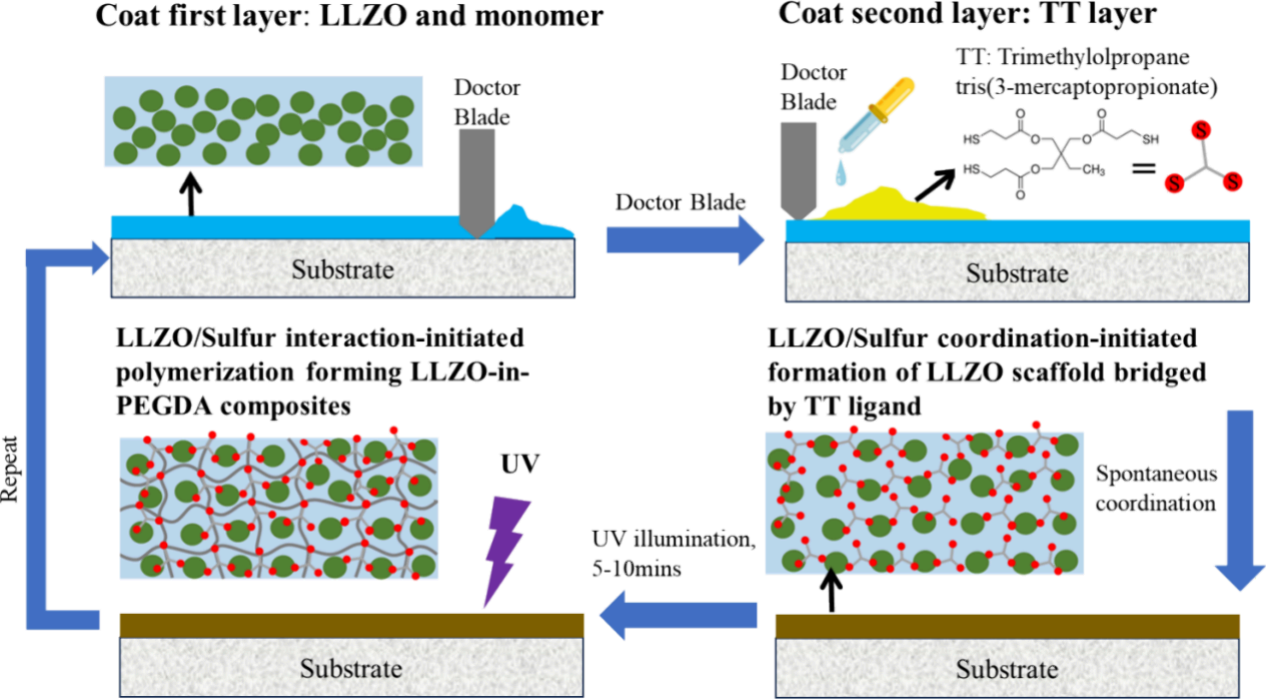
Layer-by-Layer fabrication of LLZO-in-PEGDA composite SEs via metal/sulfur interactions.

The metal/sulfur interactions (i.e., coordination interaction and electron-transfer interaction) create a sulfur-containing interphase layer on the surface of LLZO nanoparticles, which will impact the Li-ion-transport pathways around and across the LLZO phase. To identify the impact of sulfur-containing layer on Li-ion transport, the weight percentage (wt %) of LLZO and PEGDA components was varied. In addition, the highly conductive IL electrolyte EMIM-TFSI was incorporated into selected composites to enhance the number of conductive pathways available for Li-ions, thereby enhancing overall ionic conductivity.

The kinetics of the photopolymerization of the LLZO-in-PEGDA composite assisted by metal/sulfur interactions was monitored by comparing the acrylate group and thiol group before and after UV irradiation via FTIR spectroscopy. Figure 5a shows the FTIR spectra of each precursor, including raw LLZO, TT, and PEGDA monomer, as well as a representative LLZO-in-PEGDA composite, which contains 10 wt % LLZO, denoted as polymer/LLZO_10_ composite. The simultaneous decrease in the intensity of both the thiol twisting vibration band at 2570 cm^–1^ and the acrylate double-bond twisting bands at 1640 and 810 cm^–1^ indicates that both functional groups are participating in the reaction.^27–29^ Similar phenomena were observed in a representative control sample with 0 wt % LLZO, 50 wt % IL, and 50 wt %PEGDA (denoted as polymer_50_IL_50_); see Figure S4. Notably, no exogenous photoinitiators were required for the formation of LLZO-in-polymer composites via metal/sulfur interaction-initiated photopolymerization. In contrast, photoinitiators are required for the photopolymerization of control samples lacking LLZO or TT. Figure 5b presents the FTIR spectra of polymer/LLZO_10_ composites fabricated (i) with or without a photoinitiator in the presence of TT and (ii) with or without TT in the presence of a photoinitiator. The consistent peaks across these spectra confirm our previous assumption that metal/sulfur interactions facilitate the generation of key radicals, such as thiyl and carbon radicals, which are crucial for initiating photopolymerization.

**Figure 5:**
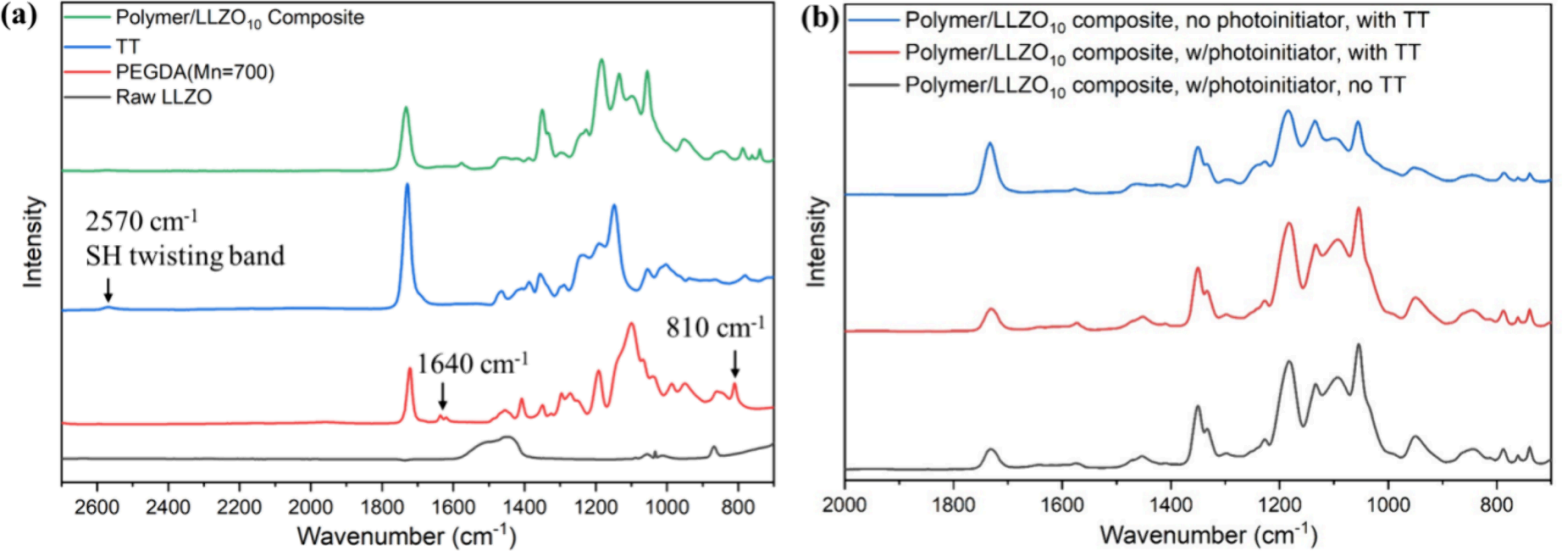
(a) FTIR spectra of a typical polymer/LLZO_10_ composite via metal/sulfur-initiated photopolymerization, as well as each individual precursor for forming the composite; (b) FTIR spectra of polymer/LLZO_10_ composites were fabricated (i) with or without a photoinitiator in the presence of TT and (ii) with or without TT in the presence of a photoinitiator.

Due to the fast kinetics of metal/sulfur interactions, the layer-by-layer fabrication method enables precise control over the structure–property relationships of LLZO-in-polymer composites by forming a planar structure with predefined composition, homogeneity, and thickness. In contrast, directly mixing the precursor solutions and TT in a bulk solution, as demonstrated in section 4.1, results in solidified products with poor control over the homogeneity and structure of the final product. This comparison is illustrated in Figure S5.

### 5.2 Impact of Metal/Sulfur Interactions on Li-Ion Transport

The various intrinsic interphases within LLZO-in-PEGDA composites play a key role in determining the true ion-transport pathways and Li-ion conductivity. The incorporation of an IL incorporates a new phase within the LLZO-in-PEGDA composites, providing a more conductive pathway. The coordinated TT creates a sulfur-containing layer on the surface of LLZO, which will impact the Li-ion transport across the LLZO|LLZO, LLZO|PEGDA, and LLZO|IL interphases. Figure S6 shows the conductivity of the composites contain solely PEGDA polymer scaffold loaded with IL, denoted as polymer/LLZO­(0 wt %)/IL. The incorporation of TT into the polymer scaffold consistently resulted in higher ionic conductivity, with the highest conductivity reaching 2.5 × 10^–3^ S cm^–1^ when loaded with 75 wt % IL. This increased ionic conductivity can be attributed to the transition to thiol acrylate mixed-mode polymerization,^29^ resulting in a soft and flexible polymer scaffold with lower Young’s modulus, as demonstrated in Zhong’s work.^29^ Therefore, the PEGDA polymer scaffold incorporated with TT exhibited a larger mesh size and a smaller interphase area, as depicted in Figure S7. The larger polymer mesh size influences Li-ion transport pathways by preferentially incorporating the more conductive IL phase.

A similar enhancement of ionic conductivity in the presence of TT was observed in LLZO-in-PEGDA composites at varying loading percentages of LLZO. In Figure 6a, when LLZO-in-PEGDA composites are loaded with 25 wt % IL, ionic conductivity increases as LLZO amount increases from 10 to 30 wt %. The conductivity reaches 5.1 × 10^–4^ S cm^–1^ in the presence of TT with 30 wt % LLZO as the active filler, comparable to or even exceeding the vendor-reported bulk ionic conductivity of LLZO (5.0 × 10^–4^ S cm^–1^). A similar trend was observed in LLZO-in-polymer composites loaded with 0 wt % IL, as shown in Figure 6b. The observed enhancement in ionic conductivity when TT is incorporated in both samples suggests that the sulfur-containing layer on the surface of LLZO promotes faster Li-ion transport across the intrinsic LLZO/LLZO and LLZO/PEGDA interphases. The further increase in ionic conductivity beyond that of bulk LLZO is primarily attributed to the incorporation of the highly conductive IL phase, which provides efficient transport pathways and reduces the reliance on the more resistive PEGDA matrix. Additional improvements in conductivity may be achieved by increasing the IL content, provided that the balance among all components is carefully maintained to preserve the composite’s mechanical integrity. The comparison of detailed EIS spectra can be found in Figure S8a,b.

**Figure 6:**
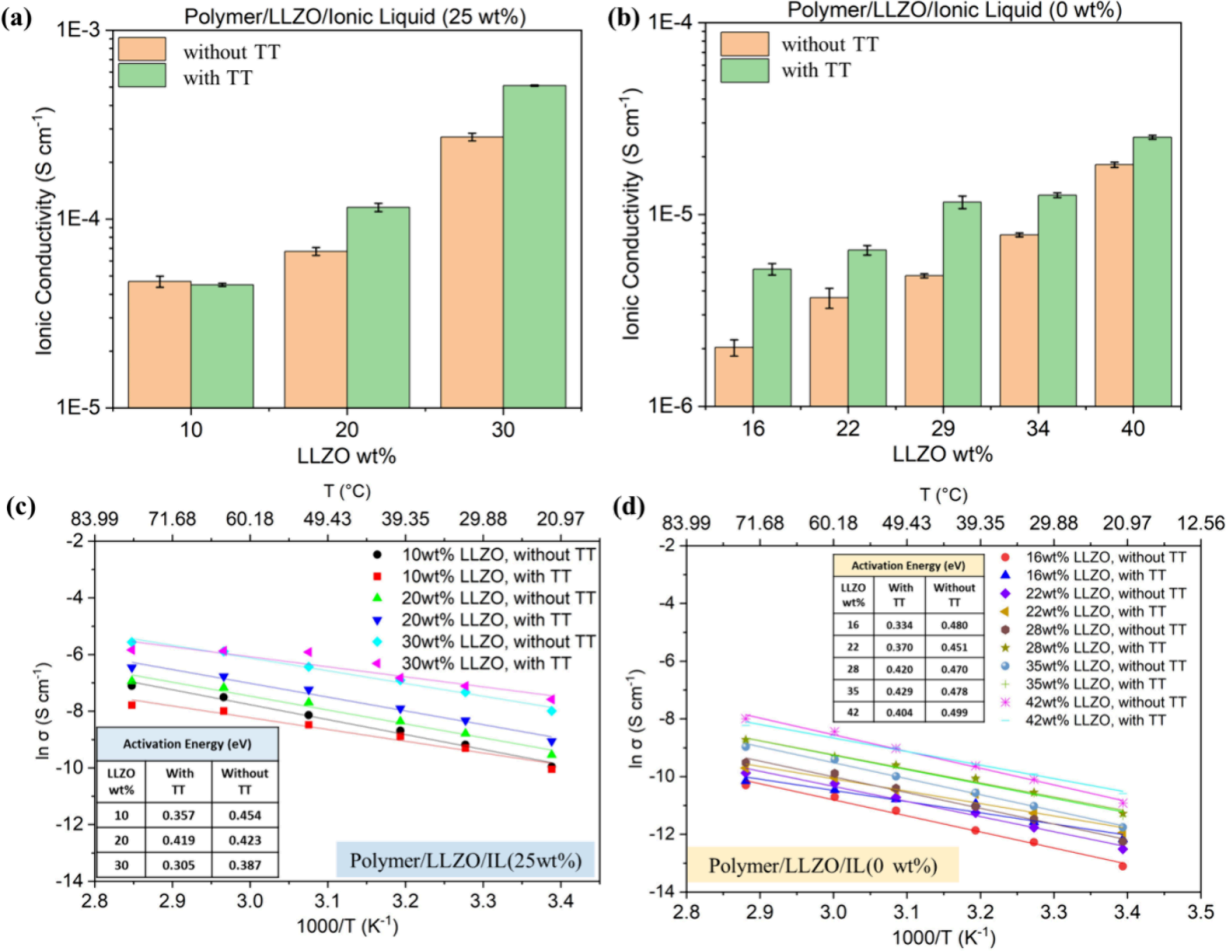
(a,b) Ionic conductivity of LLZO-in-polymer composites formed with and without sulfur-containing TT, loaded with 25 and 0 wt % IL, respectively. (c,d) Temperature-dependent ionic conductivity values of LLZO-in-polymer composites formed with or without TT and loaded with 25 and 0 wt % IL, respectively.

To further understand the role of sulfur-containing layers, we replaced the active filler with an inactive filler, such as TiO_2_. The ionic conductivity comparison between active and inactive fillers is shown in Figure S9. At high filler loading (i.e., 30 wt %) when active LLZO starts to contact, the reduced LLZO/LLZO interfacial resistance due to the sulfur-containing layer is beneficial for the formation of a percolated network, leading to a further increase in ionic conductivity. However, due to the large resistance in the bulk of TiO_2_ and at the TiO_2_/TiO_2_ interface, there is no such percolated network, leading to further saturation or even decrease in ionic conductivity.

To further elucidate the enhanced ionic conductivity, temperature-dependent ionic conductivity measurements were performed in the range of 20 to 80 °C. The activation energy (*E*_a_) of the composites was calculated according to the Arrhenius equation:

$$\sigma (T)=\sigma_{0}\exp(-\frac{E_{a}}{kT})$$ 2

where σ­(*T*) is the ionic conductivity at absolute temperature *T* (K), σ_0_ is the pre-exponential factor, and *k* is the Boltzmann constant, with a value of 8.617 × 10^–5^ eV/K.

The temperature-dependent ionic conductivity of LLZO-in-PEGDA composites formed with and without TT and loaded with 25 and 0 wt % IL is shown in Figure 6c,d, respectively. The calculated *E*_a_ values as a function of LLZO wt % for these composites are provided in the inset tables within the figures. The composites formed with TT consistently exhibit lower *E*_a_, suggesting that the sulfur-containing TT layer may promote rapid Li-ion association and dissociation at various intrinsic interphases, including LLZO/LLZO, LLZO/PEGDA, and LLZO/IL intrinsic interphases.

Figure S10 presents the SEM and energy-dispersive X-ray spectroscopy (EDS) results of a representative sulfur-containing composite (i.e., polymer/LLZO (20 wt %)/IL (25 wt %)). The LLZO nanoparticles were homogeneously distributed in the LLZO-in-polymer composites, showing no signs of phase separation. Notably, EDS analysis revealed a sulfur-to-Zr atomic ratio of 3.6:1 when the optimized TT solution, diluted with CH_2_CL_2_ at a 1:1 volume ratio, was applied during the layer-by-layer fabrication process. This suggests that each Zr atom was likely coordinated with 3–4 sulfur atoms in sulfur-containing LLZO-in-PEGDA composites.

The stress–strain curves of the representative LLZO-in-PEGDA composites show a significant difference for samples with TT fabricated by this layer-by-layer method (Figure 7). The table inset compares the values of strength, elongation at break (%), and toughness of the two composites with and without TT. With the incorporation of TT, the LLZO-in-PEGDA composite became softened, exhibiting doubled toughness (up to 2.3 J/m^3^) and a large increase in stretchability (elongation at break increased from 9 to 49%). This will result in better interfacial contact with the cells, helpful for reducing interfacial defects and achieving more uniform Li-ion flux.^21^ Such increased toughness and stretchability suggest that the sulfur-containing LLZO-in-PEGDA composites can serve a dual role as a mechanical buffer for protecting the electrode integrity during repeated volume expansion and contraction.

**Figure 7:**
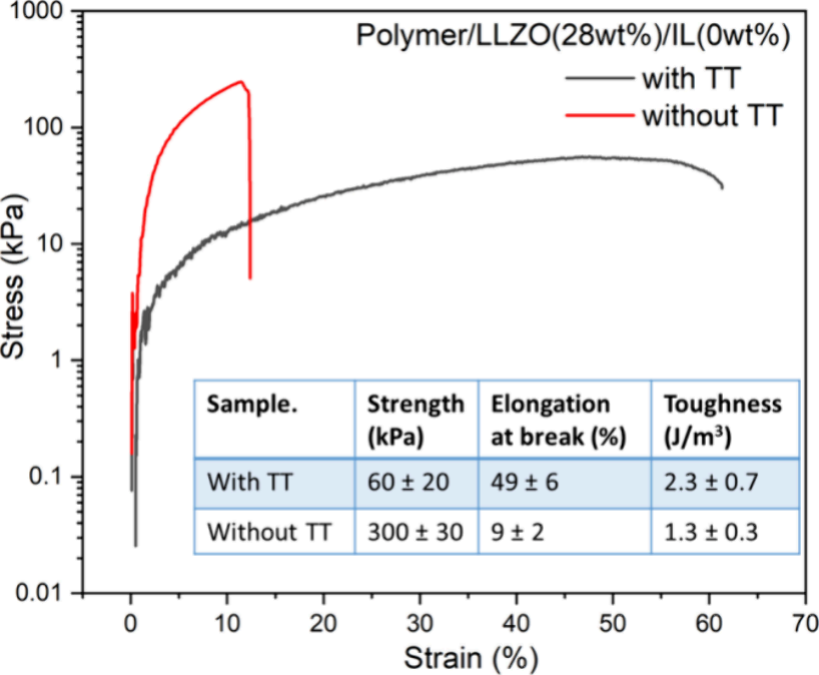
Representative tension stress–strain curves for the composites (i.e., Polymer/LLZO (28 wt %)/IL (0 wt %) composites) formed with TT and without TT.

The electrochemical stability of the LLZO-in-PEGDA composites was evaluated under the Cu|Composite|Li cell configuration by the CV method at room temperature in the potential range of −0.1 to 4 V at a sweeping rate of 0.01 V/s by using BioLogic electrochemical workstation. The selective CV plots for the sample polymer/LLZO (20 wt %)/IL (25 wt %) from second to sixth cycles are shown in Figure 8a. The CV plots remained stable under repeated scanning up to 4 V, suggesting a wide electrochemical stability window up to 4 V.

**Figure 8:**
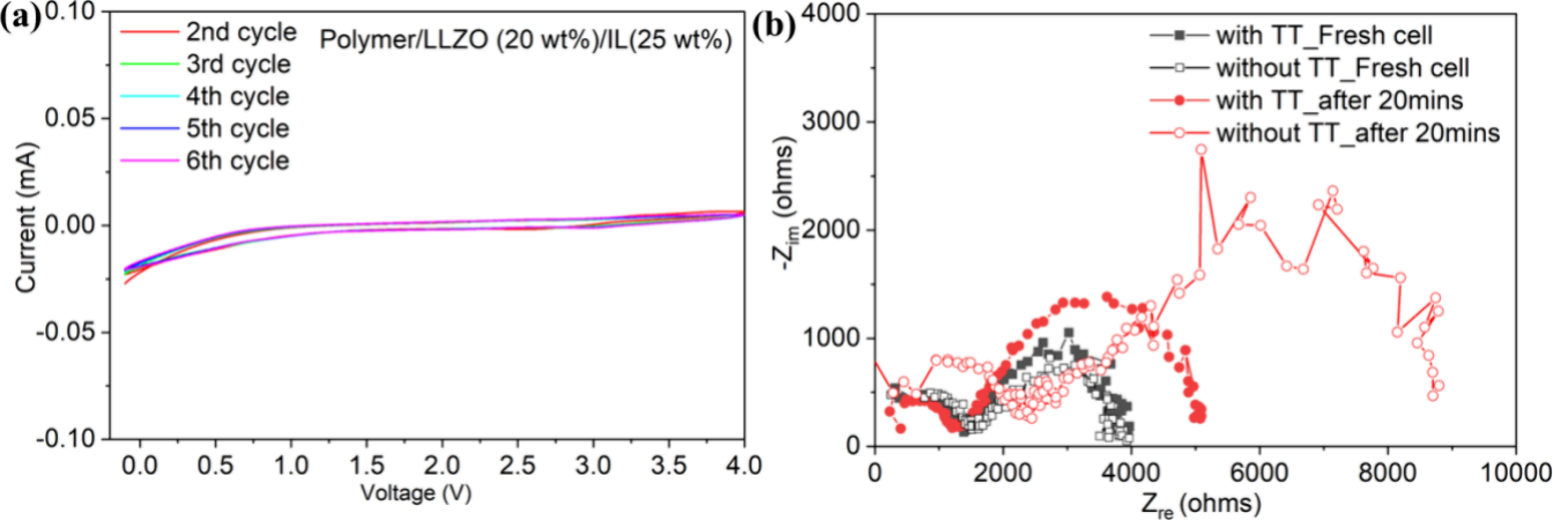
(a) CV scans for a sulfur-containing LLZO-in-polymer composite; (b) EIS comparison between the composites formed with and without TT collected on symmetrical cells after being freshly prepared and after stabilizing for 20 min.

Figure 8b compares the EIS plots of the composites formed with and without TT using the Li|Composite|Li symmetrical cell configuration. For symmetric cells, the EIS plots were collected after the cells were freshly assembled and after being stabilized for 20 min. Initially, the two cells exhibited similar bulk resistance and similar Li|composite interfacial resistance (radius of the second semicircle). After 20 min, the interfacial resistance of the composite electrolyte without TT increased significantly (red void circle). In contrast, the composite electrolyte formed with TT exhibited a much more stable interfacial resistance with Li metal, increasing to a lower value (red solid circle). The slower increase in interfacial resistance upon contact with Li-metal suggests that the presence of sulfur-containing TT on the surface of LLZO can electrochemically generate sulfur-containing organic units, such as organosulfides/organopolysulfides, simultaneously forming a stable solid-electrolyte interphase (SEI) layer on Li-metal.^48^ The sulfur-containing SEI layer is worth being the focus of future studies for preventing Li dendrite formation.

## 6 Conclusions

In summary, we uncovered unique metal–sulfur interactions between LLZO and sulfur-containing thiol groups and elucidated the underlying polymerization mechanisms initiated by the metal/sulfur interactions. Our findings revealed a preferential coordination between Zr and sulfur, accompanied by electron transfer from sulfur to Zr and La ions in LLZO upon UV activation. The metal/sulfur interactions lead to the formation of thiyl radicals capable of initiating photopolymerization of the sulfur-containing PEGDA scaffold. To harness the rapid kinetics of metal/sulfur interactions, we developed a layer-by-layer fabrication method to construct rationally designed LLZO-in-PEGDA composites with controlled composition, morphology, and structure. Notably, the LLZO-in-PEGDA composites facilitated by metal–sulfur interactions demonstrated higher ionic conductivity and lower activation energy compared to their sulfur-free counterparts. Specifically, the sulfur-containing LLZO-in-PEGDA composite, incorporating 25 wt % IL and 30 wt % LLZO, achieved an impressive ionic conductivity of 5.1 × 10^–4^ S cm^–1^. Such an enhancement in ionic conductivity suggests that the sulfur-containing layer on LLZO facilitates faster Li-ion transport across both LLZO/LLZO and LLZO/PEGDA interphases. Additionally, the sulfur-containing LLZO-in-PEGDA composites showed improved toughness and stretchability, suggesting a potential dual role as a protective layer for electrode materials. Finally, the sulfur-containing LLZO-in-PEGDA composites exhibited a more stable interfacial contact with the Li metal, highlighting their potential for solid-state battery applications.

## Supplementary Material





## Notes

Pictures of the LLZO and monomer precursor solution; picture of the solidified products before being added into common solvents; Raman spectra of ZrO2 before and after treating with sulfur-containing TT at different S to Zr ratio; transmission FTIR spectra of a typical polymer composite; pictures of the formed composites due to metal/sulfur interactions; ionic conductivity comparison of three groups of polymer composites; polymer network; EIS spectra of LLZO-in-polymer composites; ionic conductivity comparison of active filler and inactive filler; SEM image, corresponding EDS spectra, and EDS mapping (PDF)
Mixing LLZO with TT (MOV)
