# Supplementary material for: Ceramic-in-Polymer Composite Solid Electrolyte Enabled by Metal–Sulfur Interactions with Enhanced Li-Ion Conductivity
Source: ACS Appl Energy Mater. 2025 Jun 26;8(16):11884–95. doi: 10.1021/acsaem.5c01010 (PMC12381823; doi:10.1021/acsaem.5c01010)
Supplement: Supplementary file 1 [file ae5c01010_si_001.pdf]

Supporting Information

Ceramic-in-Polymer Composite Solid Electrolyte Enabled by Metal-Sulfur Interactions with Enhanced Li-ion Conductivity

Beibei Jiang,[a] Zhantao Liu,[b] Hailong Chen,[b] Yiming Zhang,[c] Vladimir V. Tsukruk,[c] Junjun Hu,[a] Zhiming Qiang,[a] Manika Tun Nafisa,[a] Benjamin Klein,[a] and Tara Joshi[a]*

a. Department of Electrical and Computer Engineering, Kennesaw State University, Marietta, Georgia, 30060, USA

b. Department of Mechanical Engineering, Georgia Institute of Technology, Atlanta, Georgia, 30332, USA

c. Department of Materials Science and Engineering, Georgia Institute of Technology, Atlanta, Georgia, 30332, USA

* Corresponding author, email: bjiang1@kennesaw.edu

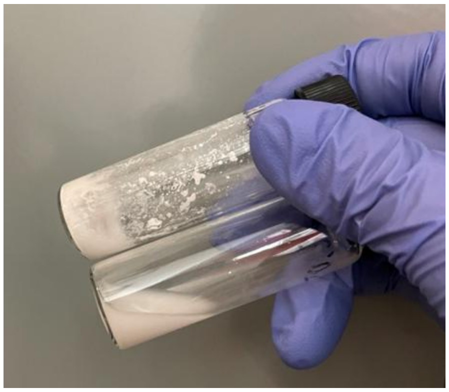

Figure S1. Pictures of the LLZO and monomer precursor solution with (Top vial) and without (Bottom vial) adding the sulfur-containing Trimethylolpropane tris(3-mercoptopropianate) (TT) solution. The bottom vial represents the liquid-state mixture. The top vial shows the solidified products formed immediately after contact between LLZO and TT.

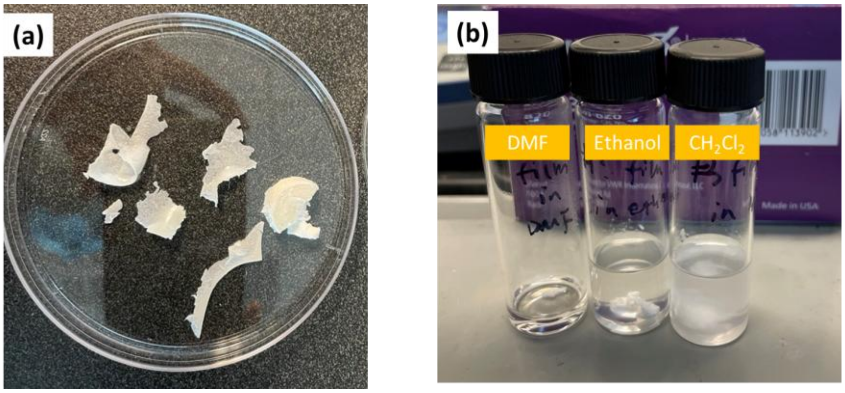

Figure S2. (a). Picture of the solidified products before being added into common solvents. (b). Picture of the products remain unchanged in three common solvents (DMF, Ethanol, and CH_2Cl_2) after 24 hours, suggesting the polymerization process of monomers or the formation of strong bonding or interaction in the products.

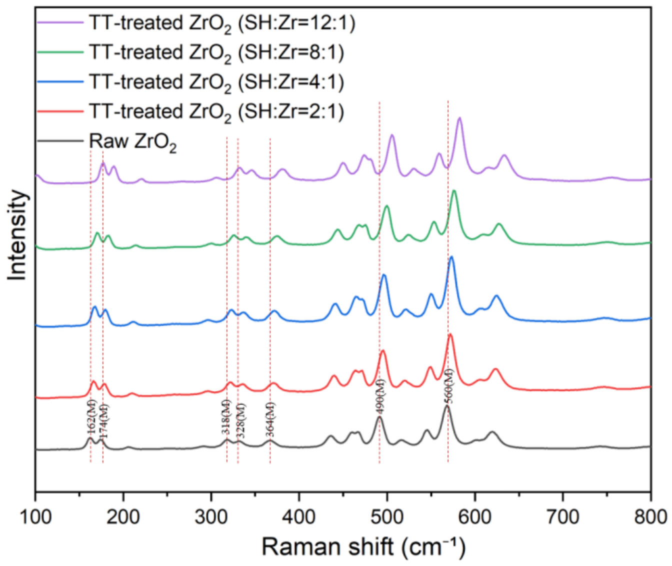

Figure S3. Raman spectra of ZrO_2 before and after treated with sulfur-containing TT at different S to Zr ratio. The distinct vibrational bands observed at 162, 174, 318, 328, 364, 490, and 560 cm^{-1} were attributed to the vibrational modes of the monoclinic phase of ZrO_2 (M). The systematic shifting toward higher wavenumber was observed when increasing sulfur content, suggesting the Zr-O bonds become shorter when coordinated with sulfur.

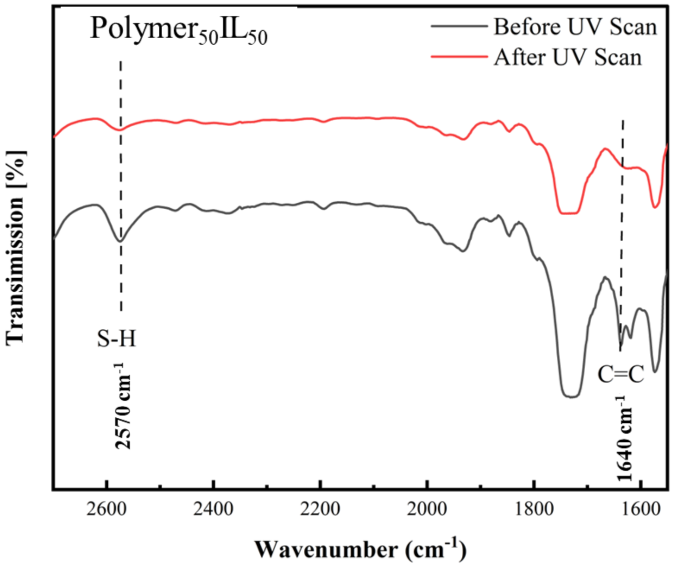

Figure S4. Transmission FTIR spectra of a typical polymer composite containing 50wt% PEGDA polymer scaffold and 50 wt% Ionic Liquid (IL). The peaks at 1640 cm^{-1} vanished completely, suggesting the full conversion of acrylate C=C bond. The peak at 2570 cm^{-1} is not fully vanished, suggesting some unreacted thiol groups.

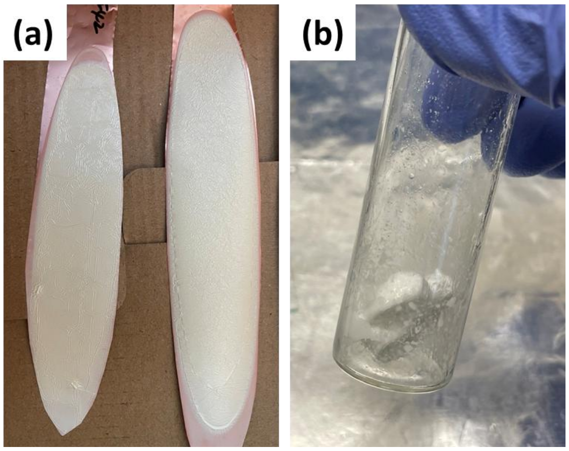

Figure S5. Pictures of the formed composites due to metal/sulfur interactions when (a). LLZO and PEGDA precursors are pre-shaped in a 2D planar film format. The films are composed of 6 layers in total, including alternating LLZO+PEGDA layer and subsequent TT layer. The total film thickness is 225±5um, which is very close to the total thickness of the 3 layers of LLZO+PEGDA (100+50+50=200um); and (b). LLZO and PEGDA precursors are in a 3D bulk format.

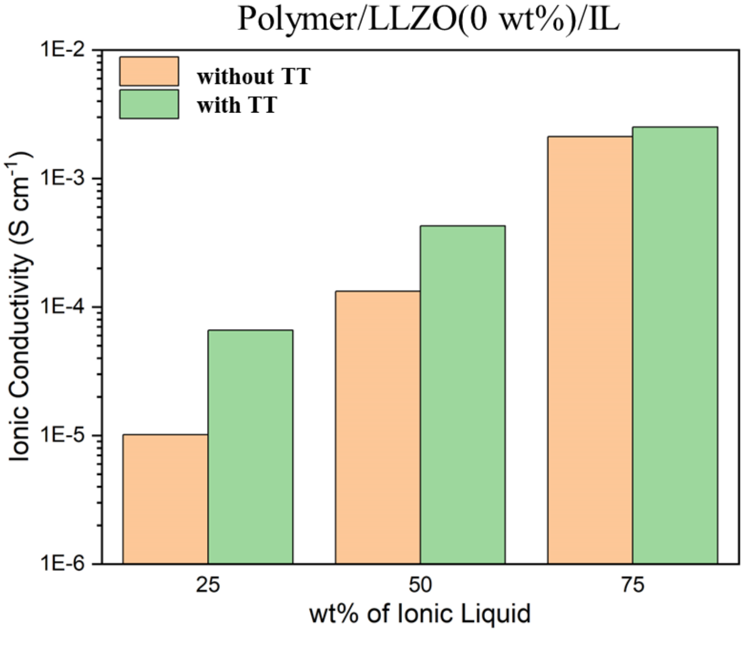

Figure S6. Ionic conductivity comparison of three groups of polymer composites formed with and without adding TT. For each group, the composites contain solely PEGDA polymer scaffold loaded with TT, denoted as Polymer/LLZO(0 wt%)/IL.

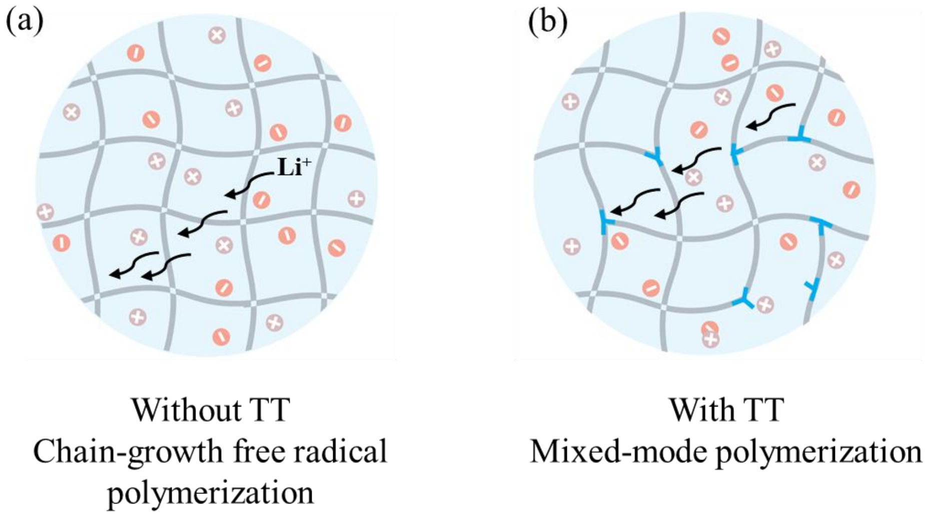

Figure S7. Illustration of Polymer network obtained from (a). chain-growth free radical polymerization in the absence of TT; and (b). mixed-mode polymerization in the presence of TT.

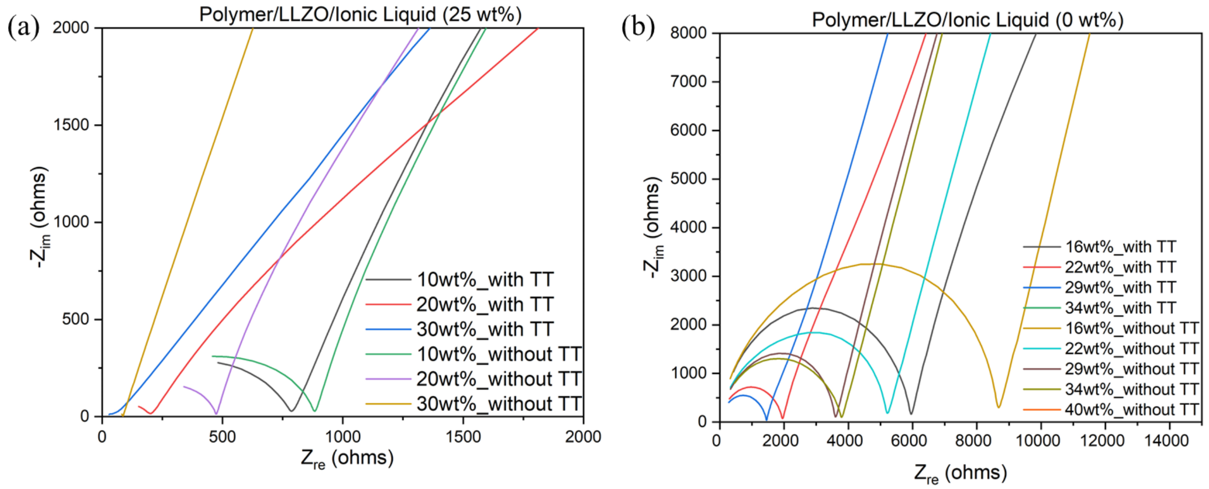

Figure S8. EIS spectra of LLZO-in-Polymer composites formed with and without sulfur-containing TT, loaded with (a). 25 wt% IL; and (b). 0 wt% IL

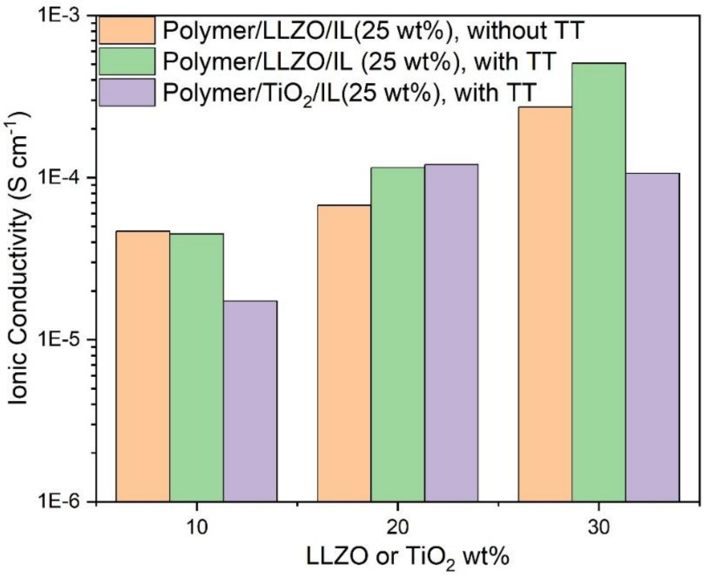

Figure S9. Ionic conductivity comparison of active filler and inactive filler (TiO_2).

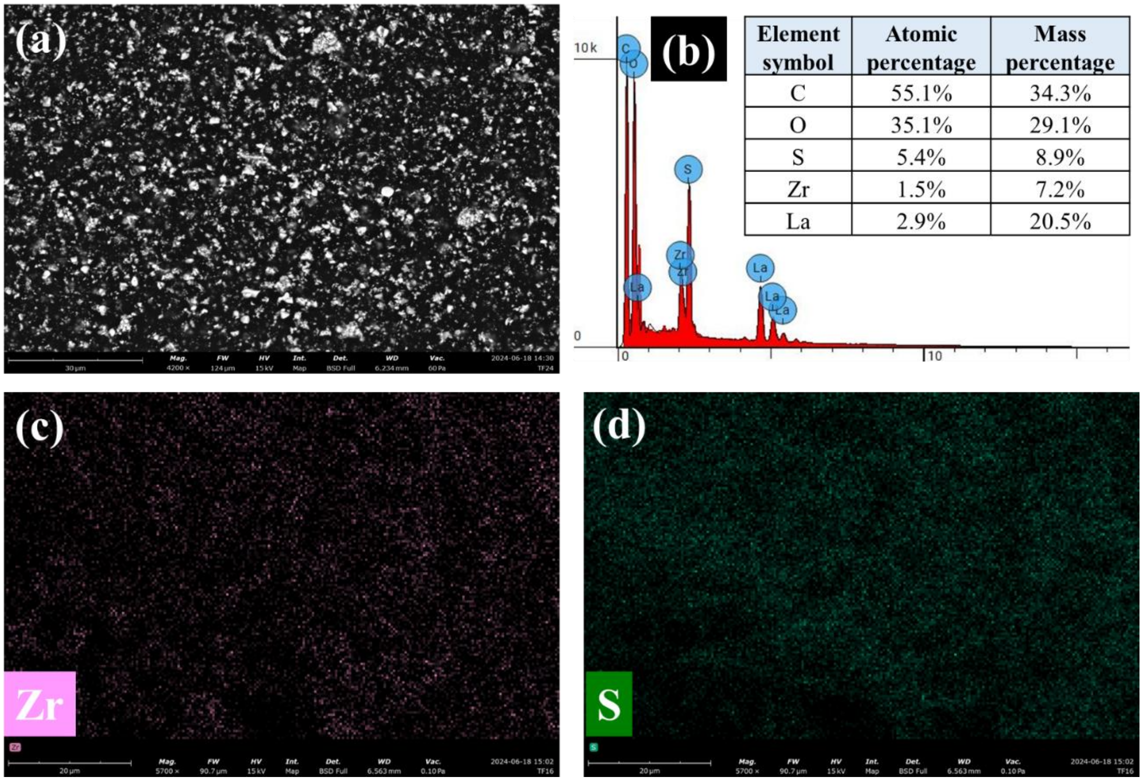

Element symbol	Atomic percentage	Mass percentage
C	55.1%	34.3%
O	35.1%	29.1%
S	5.4%	8.9%
Zr	1.5%	7.2%
La	2.9%	20.5%

Figure S10. (a). SEM image for a sulfur-containing LLZO-in-Polymer composite (Polymer/LLZO(20wt%)/IL(25wt%)); (b) the corresponding EDS spectra; (c). EDS mapping for Zr atom; and (d) EDS mapping for sulfur (S) atom.
